# Therapeutic Notes

**Published:** 1896-07-11

**Authors:** 


### Therapeutic Notes.

### OREXIN AS A GASTRIC STIMULANT AND STOMACHIC.

An interesting controversy centred round this drug during the early nineties, but during the last few years interest in its efficacy seems to have waned. Attention has, however, been forcibly drawn to this drug by a recent paper by Holms, of Wiesbaden.[1] Chemically orexin must be regarded as phenyl-dihydro-chinazolin; it is a whitish powder with a pungent taste. It has basic properties, and is practically insoluble in water, although its hydrochloride, a more irritating body, is fairly soluble in aqueous media. It is usually given in doses of 4—10 grains. Penzoll,[2] in 1890, was the first to draw attention to this drug, and he claimed for it unique properties as a true stomachic or appetite producer (ὄρεξις—appetite). In conditions of failing appetite, as in pulmonary tuberculosis, anæmia, pregnancy, &c., its administration was stated by the author to be followed by improvement in the appetite and increased bodily weight. In the same year Martin[3] repeated Penzoll's experiments, checking his results by control observations with inert substances. His conclusions were, however, adverse to the general employment of the drug, seeing that he could obtain no indisputable evidence that orexin either improved the appetite or general condition. On the other hand, in 1892 Rizzi[4] obtained striking results in support of Penzoll's claims for the drug, and suggested that previous failures might have been due to impurities in the preparation, or to the selection of unsuitable cases. Such conditions as gastralgic hyperæsthesia of the gastric mucous membrane were to be regarded as contra-indications for its employment. The most favourable subjects, according to his observations, were those afflicted with atonic dyspepsia and anæmia. He recommended its administration in cachets to be followed by a copious draft of fluid, in order that the entire mucous membrane might be flushed with a dilute solution, instead of parts of it being damaged by the concentrated form when this precaution was neglected. The more scientific and exact experiments of Hemme[5] substantiated the foregoing clinical deductions, and proved that orexin was really a gastric stimulant. His method was to extract a portion of the gastric juice after the administration of various gastric stimulants, and to analyse the product thus obtained. He made use of such bodies as hydrochloric acid and pepsin, pancreatic extract and alkalis, guaiacol, pepper, and orexin, and, as far as orexin was concerned, he proved that it had very decided powers of increasing the quantity and the activity of the gastric secretions. For the vomiting and anorexia of pregnancy orexin has been highly spoken of, and in the hands of Frommel it has been particularly successful. About the same date as the latter communication Penzoll[6] replied to his critics, and collected all the reported cases in which orexin had been tried since its introduction three years previously. He claimed that most of the evidence was highly favourable to the drug, and with regard to the failures he attributed these results to want of perseverance and the limited time allowed for the drug to manifest its advantages. The latter appears a rather weak contention, as in his first communication he claims rapid results, and a gastric stimulant, to be of use, should not only act by accumulation. With more force it might have been urged that the samples of the drug used were impure, or the cases selected unsuitable.

Holms's[7] communication of the present year should certainly rekindle interest in this drug, since his experiments go to show that a certain proportion of cases of anorexia complicating various conditions, showed distinct improvement with increased appetite and gain of weight after a course of treatment with orexin. Twenty-one out of thirty-three cases treated with good results, and only two were absolute failures.

[1] Therap. Monals., Jan., 1896. [2] Therapeut. Monals., Feb. 1890. [3] Pharm. Journal, May 31, 1890. [4] Gazz. Med. Lombard, June 18, 1892. [5] Zeitschaft für Klin. Med., Bd. xix. [6] Centralblatt für Gynäk, No. 16, 1893. [6] Therapeut. Monals., May, 1893. [7] Therapeut. Monals, Jan., 1896.

---

### THE USE OF NASCENT OXYGEN IN MEDICINE.

Nascent oxygen has frequently been employed as a therapeutic agent, either in the form of ozonic ether, peroxide of hydrogen, pyrozone, oxygenised turpentine, aquozone, and, more recently, as ozonised air. It has been employed[1] with success in cases of typhoid, asphyxia, tetanus, and for hydrophobia its administration has been recommended. H. S. Norris[2] attaches value to its oxidising powers in cases of pulmonary phthisis. He administers it in the form of "aquozone," which is a solution of hypophosphites in which ozone is present to the extent of about 2½ per cent.; the addition of the hypophosphites is to maintain the stability of the solution. The quantity to be given should be about 3 oz. four times a day, before each meal and before going to bed. It appears to have been of value in those cases in which the patient was under thirty years of age; when the disease was advanced in the second stage; and when the phthisis was of the catarrhal variety, and not too rapid in its advance. If both lungs were attacked, and the area of disease extensive, the method appeared to be less successful.

R. J. Cowen[3] has recently recommended the employment of ozonised air in cases of leucocythæmia. The patient is allowed to inhale the air through a specially prepared mask, which is in connection with a modified Andriolis apparatus. The air, by means of the passage of the electric spark, is charged to the extent of 75 per cent. with ozone. At first oppression and giddiness is complained of, but after two or three sittings no objections are raised on these grounds. The general condition appears to improve under this treatment; the number of white corpuscles in the blood decreases; the anæmia is less marked; and the size of the spleen decreases. Cowen believes that in leucocythæmia and allied conditions the hæmoglobin of the blood is in an altered condition, whereby it is unable to combine readily with the oxygen of the air, and that this impaired power of the hæmoglobin for taking up and parting with oxygen is chiefly responsible for the increased number of leucocytes and other symptoms of the condition.

It occurred to him to try the effect of bringing oxygen in the form of ozone in contact with the hæmoglobin by means of the above respiratory apparatus, believing that by this means the oxidation of the hæmoglobin would be more complete, and that the system might have time given it to recover its normal energy and resume its proper function of corpuscle formation. The success which attended the case reported seems to warrant further trial of the method.

[1] Lancet, Vol. I., page 960, 1891. [2] H. S. Norris, New York Med. Jour., No. 5, 1892. [3] Lancet, 1896, Vol. I., page 1,278.

